# Towards technology, economy, energy and environment oriented simultaneous optimization of ammonia production process: Further analysis of green process

**DOI:** 10.1016/j.heliyon.2023.e21802

**Published:** 2023-11-07

**Authors:** Ashish M. Gujarathi, Rashid Al-Hajri, Zainab Al-Ani, Mohammed Al-Abri, Nabeel Al-Rawahi

**Affiliations:** a Petroleum and Chemical Engineering Department, College of Engineering, Sultan Qaboos University, Muscat, Oman; b Mechanical and Industrial Engineering, College of Engineering, Sultan Qaboos University, Muscat, Oman; c Director, Nanotechnology Research Center, Sultan Qaboos University, Muscat, Oman

**Keywords:** Green hydrogen, Green ammonia, Energy, Economics, Multi-objective Optimization

## Abstract

Ammonia is one of the most produced chemicals around the world due to its various uses. However its traditional production process is associated with high fossil fuel consumption. To avoid this, the production of green ammonia can be done, and one of the considered production methods is water electrolysis, where the hydrogen needed for the manufacturing of ammonia is produced using solar energy. In this work, multi-objective optimization (MOO) is carried out for two ammonia synthesis processes with water electrolysis. One process uses solar energy to generate electricity for the whole process (Green ammonia), while the other uses natural gas for the same purpose (non-green ammonia) on a small production scale. The process is simulated using ProMax 5.0 and MOO is done using Excel-based MOO with I-MODE algorithm. Several MOO cases are solved with different objectives like CO_2_ emissions and energy (ENG) minimization, and Profit and Purity maximization in two and three objective cases. To conduct the work, several decision variables are selected like the operating temperatures and pressures of different streams in addition to the flow rate of nitrogen and water. Some constraints regarding the purity and reactors temperature are considered as well. The obtained results showed that the profit of green ammonia process (ranges between 0.7 and 80 M$/yr) is lower compared to the non-green process (ranges between 0.8 and 4.4 M$/yr). On the other hand, huge CO_2_ emissions (up to 38000 tons/yr) are produced in the non-green process compared to almost zero emissions with the green process. In most cases, water and nitrogen flow rates showed a high influence on the results and caused conflict between the objectives.

## Graphical abstract

**Figure:**
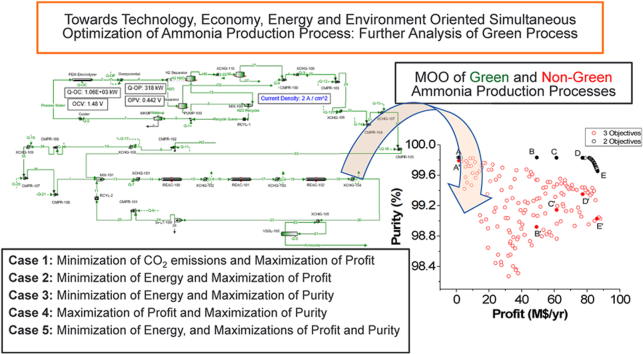

## 1 Introduction

There are several chemicals that are considered widely very important and essential due to their contribution to manufacturing other main chemicals and products and one of these chemicals is ammonia (NH_3_). The majority of ammonia production is used for producing fertilizers such as ammonium nitrate, and urea and it can be also used to manufacture paper, fibers, plastic, etc. [[1], [2], [3]]. In addition, ammonia can be used in building and industrial systems for refrigeration purposes and in power generation using fuel cells [4]. There is a constant growth in ammonia production as in 2012 it reached 137 million tons and in 2018, it increased to 140 million tons [5].

In ammonia production plants, usually, fossil fuels like coal and natural gas are being used as feedstock, as a hydrogen source, consuming about 1.2 % of the global primary energy and contributing to 0.93 % of global greenhouse gas emissions [6]. The hydrogen derived from the selected fossil fuel reacts with nitrogen (Haber-Bosch process) under high temperature and pressure operating conditions [1]. This exothermic reversible reaction considerably releases high amounts of heat and the production is highly affected by the operating conditions of the reactor and the flow rates of the gases [7]. Other more green alternatives can be used as hydrogen sources for ammonia synthesis reactions like biomass through gasification. Several countries are considering the biomass production route like Canada, Sweden, Poland, the UK, and Switzerland that use wood chips [[8], [9], [10]]. The use of biomass produces rich hydrogen gas with less carbon emissions [11]. Another process was also designed (coke-oven gas) to minimize the emissions, by capturing it, and to maximize the exergic efficiency [12].

Hydrogen can also be produced using water electrolysis in an electrolyzer that electro-thermally decomposes water to hydrogen and oxygen. Different processes are available for water electrolysis like alkaline electrolysis and polymer electrolyte membranes (PEM) electrolysis, and high-temperature solid-oxide electrolysis (SOE). The first two processes operate at low temperatures while the latter operate at relatively higher temperatures [[13], [14], [15]]. PEM electrolysis is more flexible with faster dynamic response and higher efficiency with a compact design compared to alkaline electrolysis [16].

In the PEM electrolyzer, there are two terminals, cathode and anode, with a polymer electrolyte membrane in between. Water reacts to produce oxygen gas, electrons (e^−^), and hydrogen protons (H^+^). At the cathode, electrons combine with hydrogen protons to form hydrogen gas. The overall reaction of water electrolysis can be expressed by the following [[17], [18], [19]]:

$${2H}_{2}O\left(l\right)↔{2H}_{2}\left(g\right)+O_{2}\left(g\right)$$

The source of the electricity that can be used for electrolysis and in the ammonia loop can differ, as it could be a fossil fuel or a renewable resource like solar, wind, geothermal, etc. A long time ago, the use of fossil fuels became a serious concern for humankind as the prices of these fuel resources started increasing remarkably, which stimulated researchers to develop alternative fuel resources. This introduced the concept of renewable energy, which is still in the stage of improvement regarding increasing its usage [20,21]. The main concern about using fossil fuels as energy sources, in addition to the high cost, is that they are coupled with high amounts of greenhouse gas (GHG) emissions, mainly carbon dioxide (CO_2_). Using a renewable resource for generating electricity categorizes the produced hydrogen as green since no GHG emissions are produced for its production. Green hydrogen is considered the most favorable medium for storing energy as the demand for hydrogen is expected to vastly increase in the near future [22]. Converting this gaseous hydrogen into another liquid energy carrier, such as ammonia or methanol, is required for easier and safer storage and transportation [23].

There is a lack of studies in the area of multi-objective optimization of ammonia synthesis studies. Multi-Objective Optimization of biomass to ammonia process specific to different feedstock to minimize the manufacturing costs and the global warming potential [11]. Hernández-Pérez et al. [24] used I-MODE algorithm to optimize the net profit and the CO_2_ emissions from ammonia production considering uncertain feedstock compositions of shale/natural gas. They observed that the increase in the value of the economic objective function is much more significant than the increase presented by the environmental objective function at the given points. The multi-objective particle swarm optimization was employed to perform the optimization of heat absorption rate and the total entropy generation rate for the ammonia production process [25]. Synthesizing ammonia with a Braun-type reactor was optimized with NSGA-II to reduce its entropy generation and maximize the exothermic rate. The selected model converts the heat generated in the reactors to electricity. The selected point from the obtained Pareto front improved the rate by 12.6 % and reduced the entropy generation to 3.4 % [26]. However, in the existing studies, the electrolysis-based hydrogen was never used to produce the ammonia. Moreover, two different electricity sources such as using solar cell, and using natural gas fuel were also never used in the past for the MOO study of the ammonia production process.

### 1.1 Novelty of work

In this work, multi-objective optimization (MOO) is carried out for two ammonia synthesis processes with water electrolysis. One of them uses solar energy to generate electricity for the whole process (Green ammonia), while the other uses natural gas for the same purpose (non-green ammonia) on a small production scale. The process is simulated using ProMax 5 and MOO is done using excel-based MOO with I-MODE algorithm [27]. The hydrogen required for the production is obtained using electrolysis and the ammonia is synthesized using the Haber-Bosch process [28]. Five MOO cases are solved with different objectives like CO_2_ emissions and energy (ENG) minimization, and Profit and Purity maximization in two and three objective cases. The decision variables include operating temperatures and pressures of different streams in addition to the flow rate of nitrogen and water. The obtained results showed that the profit of green ammonia is lower compared to the non-green one. On the other hand, huge CO_2_ emissions are produced in the non-green process compared to almost zero emissions with the green process. In most cases, water and nitrogen flow rates showed a high influence on the results and caused conflict between the objectives.

## 2 Methodology

### 2.1 Process description

**Fig. 1:**
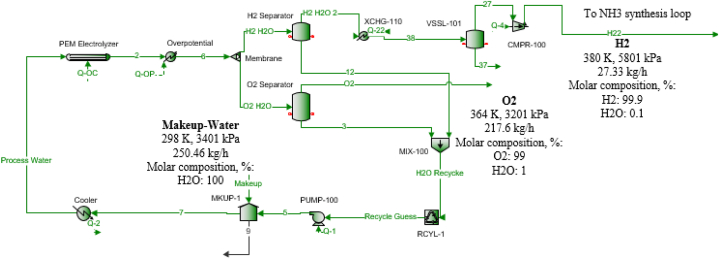
PEM electrolysis section.

**Fig. 2:**
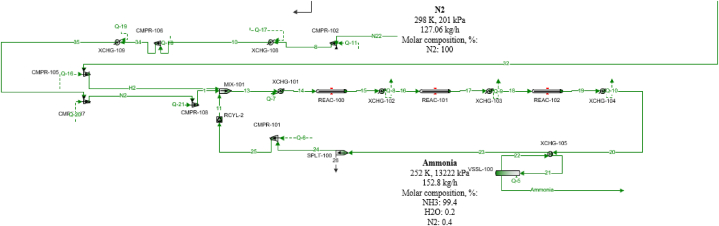
Ammonia synthesis loop section.

Ammonia production via water PEM electrolysis can be divided into two sections; the PEM electrolysis section (Fig. 1) and the ammonia synthesis loop section as seen in Fig. 2. In the PEM electrolysis section, 1339 kmol/h of water with a temperature of 363 K and pressure of 3236 kPa is cooled to 353 K to enter a PEM electrolyzer to spilt the water into oxygen and hydrogen (The overpotential unit in the flowsheet is modeled to count for the temperature change due to the electrolysis reaction). Then, a membrane is used to separate hydrogen and oxygen, where 100 % of the produced hydrogen with 50 % of the unreacted water goes into stream H_2_ H_2_O and 100 % of oxygen, and the remaining water is in stream O_2_ H_2_O. To purify the hydrogen, it goes through two steps of cooling and separation using a flash drum to remove the highest possible amount of water and produce hydrogen with 99.9 % purity that is sent later to the ammonia synthesis loop after pressurizing it to 13601 kPa using a multistage compressor with intercoolers. Oxygen is also separated from the water using a flash drum and the separated water from both oxygen and hydrogen is pumped and recycled back for cooling and electrolysis after adding the needed makeup amount of water. The nitrogen stream is assumed to be obtained readymade as a stream from an existing air separation plant. The air separation unit separating nitrogen from air is not considered in this study.

13.41 kmol/h of hydrogen that is produced from the PEM section is mixed with a pressurized (using a multistage compressor with intercoolers) 4.5 kmol/h of nitrogen. After that, the gas mixture is heated to enter the multistage ammonia synthesis catalyzed single-phase reactor with intercoolers (three stages). The catalyst used in the reactors is iron-based and to avoid its deactivation, the temperature of the outlet gas streams should not exceed 783.15 K [29]. The outlet gaseous stream (751 K) is then cooled to 450 K in a cooler and then it enters a heat exchanger to be further cooled to 316 K. To separate the produced ammonia from the unreacted gases, further cooling is needed to liquefy the maximum amount of ammonia gas with a high purity. To achieve this, a condenser is used to cool and produce liquid ammonia with 99.4 % purity at 252 K and 13222 kPa. The gas outlet from the condenser is then heated in the heat exchanger and pressurized and recycled to be mixed with oxygen and hydrogen. For the random case calculation, 27.33 kg/h of hydrogen is produced from 250.46 kg/h of makeup water added to the PEM process water stream. This shows that 9.16 kg/h of water is needed to produce 1 kg/h of Hydrogen using the existing PEM electrolyser as shown in Fig. 1. The PEM electrolyzer consumes 38.78 kWh of 1.48 V electricity per kg of hydrogen produced assuming 100 % efficiency. With 70–80 % assumed efficiency, the electricity consumption varies from 55.4 kWh to 48.48 kWh per kg of hydrogen. In this study, the electrolyzer efficiency is considered as 75 %.

The process is simulated using ProMax 5.0 and the selected package is Peng-Robinson, the used catalyst in the three-bed ammonia reactor is an iron-based catalyst and to avoid its deactivation, the temperature inside the reactor should not exceed 783 K. The well-known Haber Bosch (HB) reaction is considered for ammonia production from hydrogen and nitrogen. The HB reaction combines nitrogen with hydrogen to produce ammonia. The iron-based catalyst is used with a 40 % void fraction. The ammonia production reaction is reversible and exothermic.

$$3H_{2\left(g\right)}+N_{2\left(g\right)}↔2{NH}_{3\left(g\right)}\Delta H=-91.8kJ/mol$$

### 2.2 Problem formulation and multi-objective optimization

The main concern of any industrial process is to maximize its profit and to determine it; a detailed costing has to be done. This detailed costing comprises all the costs and sales of the industrial process including the calculation of equipment costs, construction costs, royalties, raw material and utility costs, wages for laborers, depreciation, etc. to finally calculate the profit according to the following equation [30]:

$$Profit\;before\;tax\;\left(PBT\right)=Total\;Sales\;-\;Total\;Production\;Cost$$

Minimizing the energy requirements for the process is also essential to minimize the operating cost and emissions to the atmosphere due to using electricity (in the non-green process). The total energy demand of the process can be calculated by the following equation [31].

$$ENG=\sum \left(Q_{pumps}+Q_{recompressor}+Q_{heaters}-Q_{coolers}\right)$$

where Q is the energy utilized in the unit.

The economic data consisting of capital and operating costs are shown in Table 1.

**Table 1:** Capital and operating cost details.

Parameter	Cost
Total Capital Investment ($)	78176193.86
Labor-Related Operations ($/yr)	2282800
Maintenance ($/yr)	535546.96
Operating Overhead ($/yr)	93006.81
Property Tax and Insurance ($/yr)	427014.18
Depreciation ($/yr)	3202606.40
General Expenses ($/yr)	15152.38
Energy and Utility Costs ($/yr)	11395981.47
Total Production Cost ($/yr)	17952108.23
Net Profit ($/yr)	29647016.08

The base case analysis shows that the CO_2_ emissions are at intermediate values with lesser profit for the non-green process. Their values for energy consumed kW, profit ($), and CO_2_ (ton/year) emissions are 11877, 1400379, and 23276 respectively. A more detailed comparison of CO_2_ emissions and profit is provided in section 3.1 under the case 1 discussion.

The selection of the decision variables (DV) was done based on their influence on the process performance and the objectives that are being investigated. The detailed sensitivity analysis of individual variables was conducted to check the bounds of variables and their impact on the objectives, accordingly, the ranges of DV were fixed. The flow rates of the raw materials, water (Water F) and nitrogen (N_2_ F), are affecting the amount of energy needed as well as Profit and emissions. Ever since the process has many cooling and heating units, their outlet temperature plays a significant role in this MOO study. The considered DVs and their upper and lower ranges were decided based on the capacity of the plant and after studying the sensitivity analysis of DVs on the objectives. The constraints were decided based on the literature limitations and process specifications. Table 2 capsulizes the problem formulation for the plant.

**Table 2:** Optimization cases, decision variables, and constraints for the ammonia plant.

Lower range	DV^b^	Upper range	Constraints
400	14 T, K	750	Purity > 98 % T 15, T17 and T19 < 783.15 K
400	16 T, K	750	
440	18 T, K	750	
330	20 T, K	620	
150	22 T, K	300	
1950	5 Pr, kPa	4500	
10200	1 Pr, kPa	20000	
1004	Water F, mol/h	2009	
3.40	N_2_ F, mol/h	6.80	

b # T, K : Stream # Temperature (K) ; # Pr, kPa : Stream # Pressure (kPa) ; # F, mol/h : Stream # Molar flow rate (mol/h)

The studied cases for the green-ammonia process are as below:

• Max. Profit vs. Max. Purity
• Min. Energy (ENG) vs. Max. Profit
• Min. Energy (ENG) vs. Max. Purity vs. Max. Profit

In addition to these cases, other cases are also considered for the non-green process, which are:

• Min. CO_2_ emissions vs. Max. Profit
• Min. Energy (ENG) vs. Max. Purity

## 3 Results and discussion

### 3.1 Non-green ammonia process

Case 1 : Minimization of CO_2_ emissions and Maximization of Profit

**Fig. 3:**
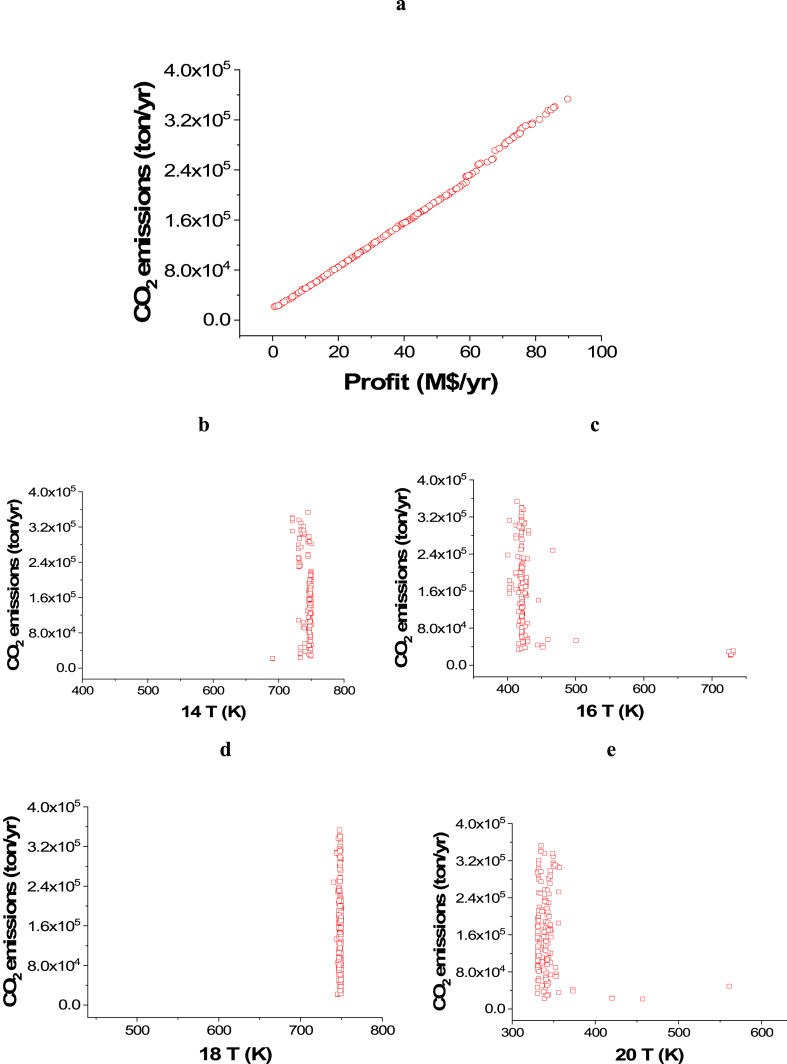

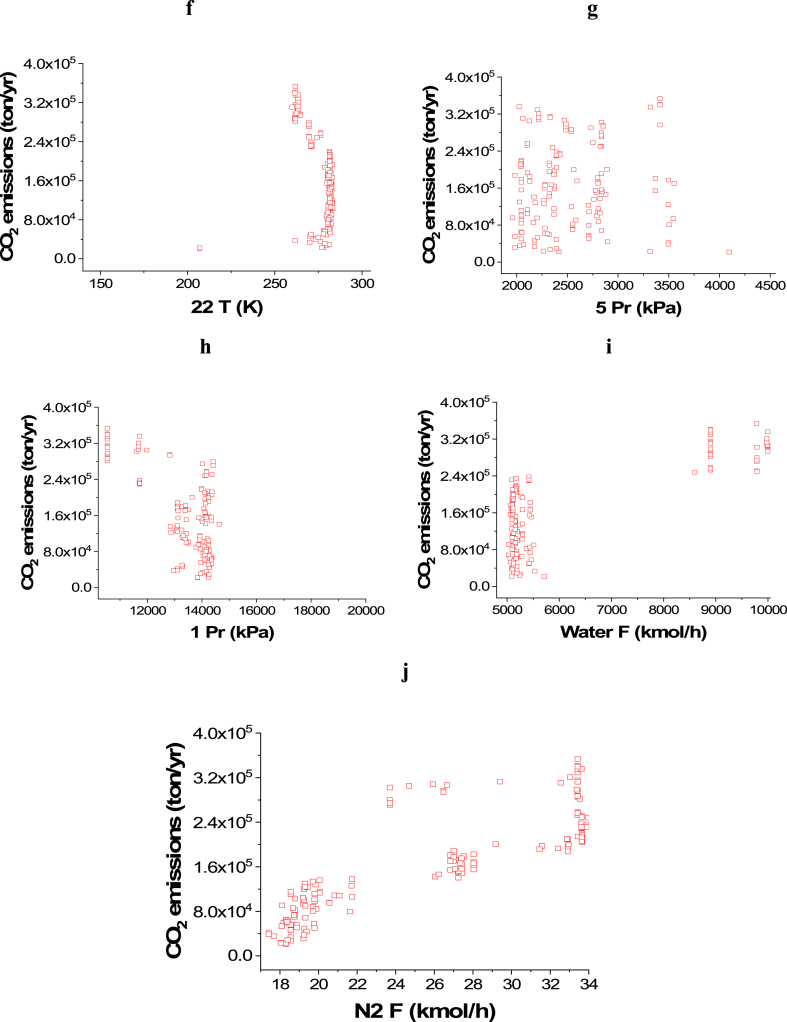
Case 1 Pareto front (a) and the corresponding decision variables (b–j) for the non-green ammonia production process (minimization of CO_2_ emissions and maximization of Profit).

The attained Pareto front for the minimization of CO_2_ emissions and maximization of Profit is shown in Fig. 3a, while the results of the corresponding decision variables are seen in Fig. 3b–j. It is noticed that the 14 T decision variable converged to the highest value in the range, which is 750 K (Fig. 3b), which is also the case with 18 T (Fig. 3d). This behavior helps in achieving the highest possible amount of ammonia, which maximizes the profit, but the CO_2_ emissions objective is not affected by these decision variables. On the contrary, 16 T (Figs. 3c) and 20 T (Fig. 3e) converged to the minimum value in the range (400 and 330 K respectively), which also happening to increase the rate of production amount and reduce the amount of refrigerant needed in the condenser where liquid ammonia is obtained. 22 T converged to values between 206 and 238 K as presented in Fig. 3f, to ensure liquefying of the maximum ammonia without using an excess amount of refrigerant. To minimize CO_2_ emissions, it can be observed that both of the pressure decision variables, 5 Pr (Fig. 3g) and 1Pr (Fig. 3h), mostly converged to the lower side of the range. Maximizing the flow rates of water and nitrogen increases power requirements for pumping and compression as displayed in Fig. 3i and j. As a result, CO_2_ emissions will increase, but the profit will also increase.

Case 2 : Minimization of Energy (ENG) and Maximization of Profit

**Fig. 4:**
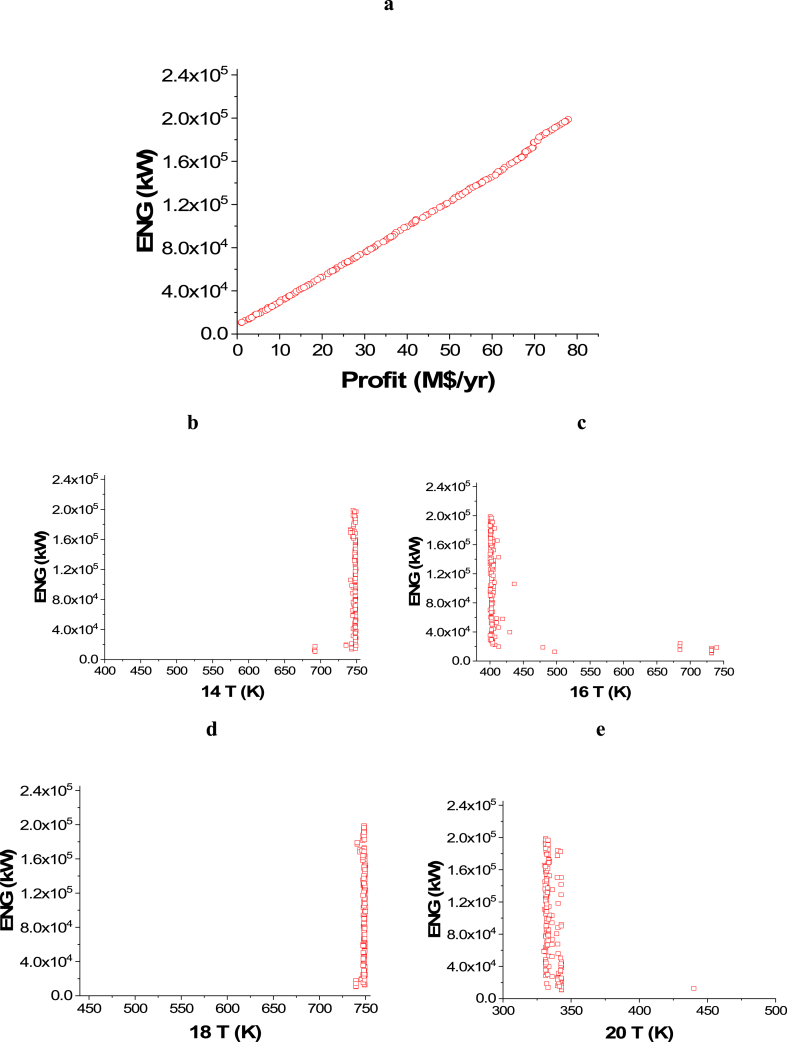

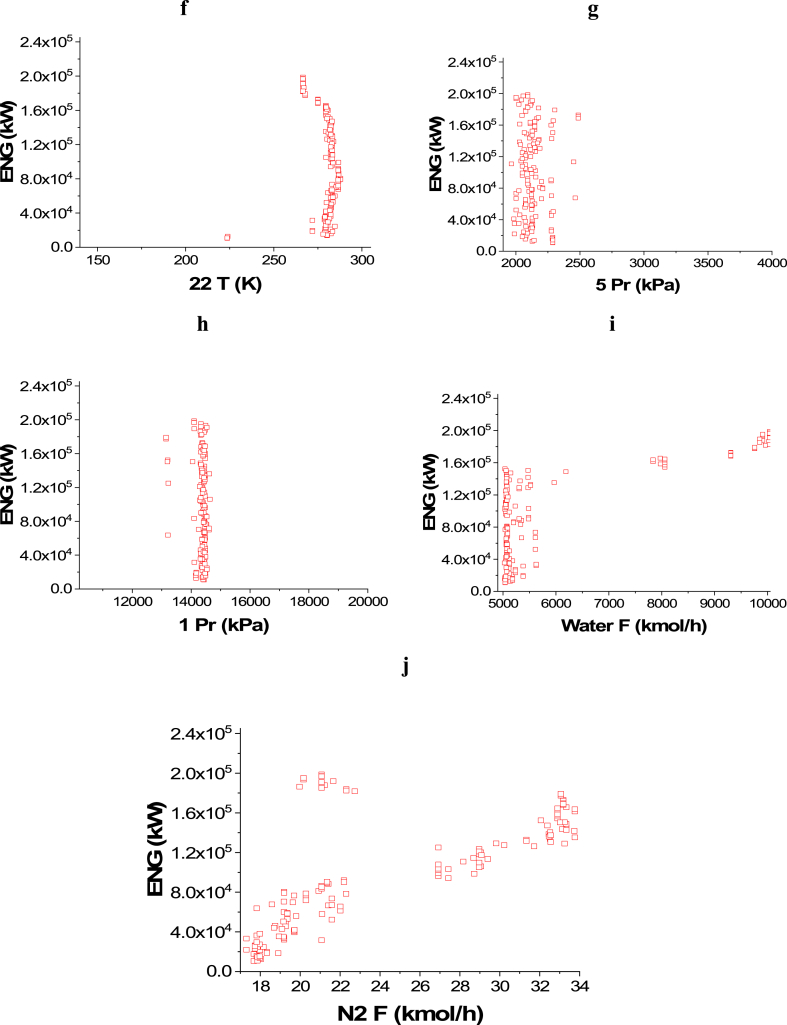
Case 2 Pareto front (a) and the corresponding decision variables (b–j) for the non-green ammonia production process (minimization of ENG and maximization of Profit).

The achieved trade-off between ENG and Profit is displayed in Fig. 4a, whereas the effects of the selected decision variables are found in Fig. 4b–j. Similar to the case of CO_2_ emissions minimization and Profit maximization, 14 T, 18 T, and 22 T converged to their upper range value as observed in Fig. 4b, **d,** and **f** respectively. On the contrary, 16 T and 20 T converged to the lower values in their ranges as shown in Fig. 4c and **e** respectively. This behavior can be explained as it reduces the amount of energy consumed, but it increases the heat credits included in the detailed cost calculations, which in turn maximizes the profit. To minimize ENG and keep a high production and profit, 5 Pr (Fig. 4g) band 1 Pr (Fig. 4h) converged to the lowest possible value. To maximize the profit, higher feed flow rates are needed, but this will result in higher energy consumption as seen in Fig. 4i–j.

Case 3 : Minimization of Energy (ENG) and Maximization of Purity

**Fig. 5:**
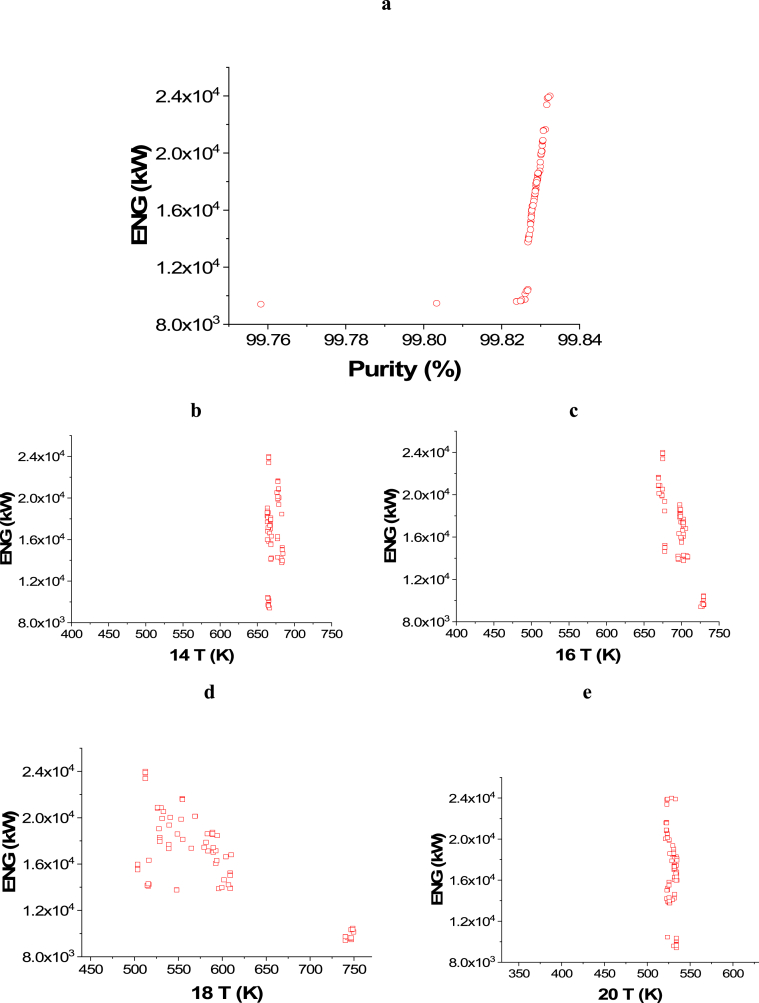

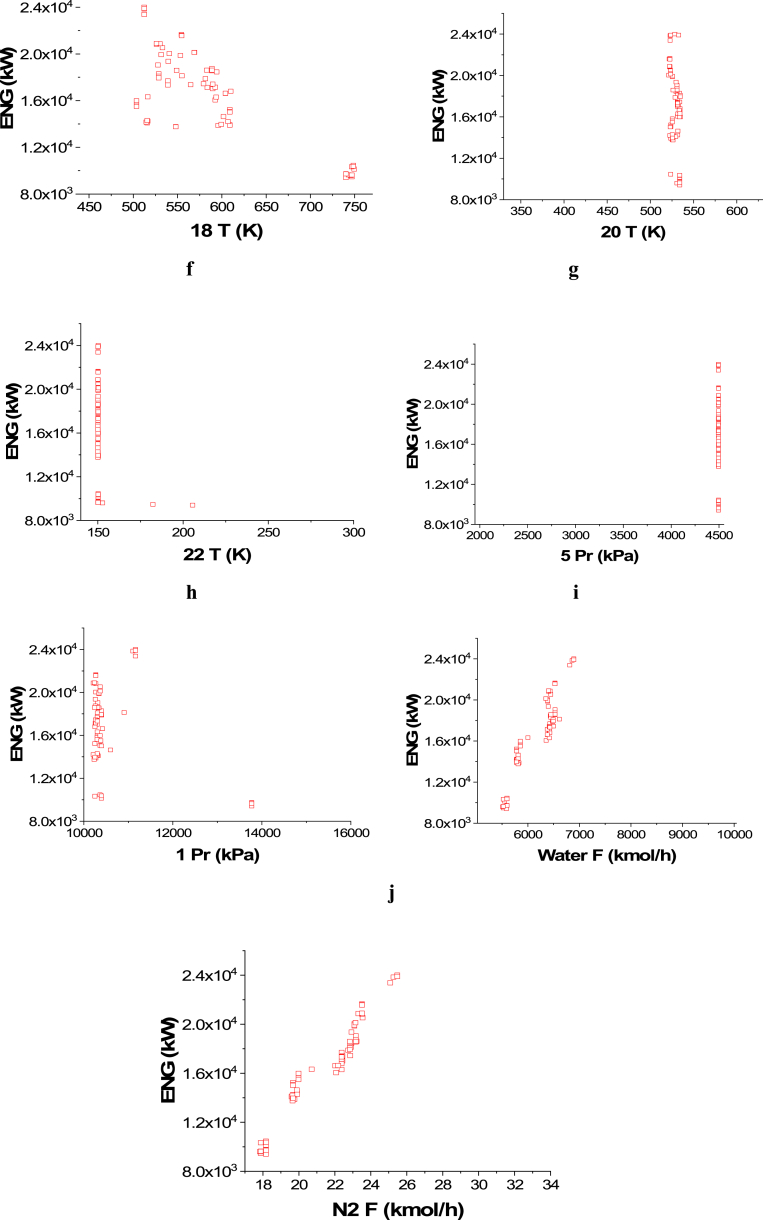
Case 3 Pareto front (a) and the corresponding decision variables (b–j) for the non-green ammonia production process (minimization of ENG and maximization of Purity).

The attained non-dominated set of solutions between ENG and Purity is exhibited in Fig. 5a, while the decision variables impacts are seen in Fig. 5b–j. To keep producing ammonia with the highest possible purity, while minimizing the energy requirements at the same time, 14 T and 16 T decision variables converged to the upper side of the range as 14 T solutions are between 650 and 700 K, while 16 T solutions are between 525 and 550 K as displayed in Fig. 5b–c. For the 18 T decision variable (Fig. 5d), it can be seen as the temperature increases, there is a reduction in ENG and this can be attributed to that, higher temperature leads to less cooling energy utilization. Similar to 18 T, 20 T results are also found around 525 and 500 K to obtain the maximum purity will minimal ENG (Fig. 5e). 22 T decision variable is not affecting ENG objective function in this optimization case, so it converged to the lowest value in the range (150 K) to achieve the maximum possible purity (Fig. 5f). To minimize the compression energy needed to pressurize the hydrogen produced before entering the ammonia loop, 5 Pr decision variable results are noticed to be converging to the upper value in the range (4500 kPa) as shown in Fig. 5g. Reducing compression pressure minimizes ENG, which explains why 1 Pr decision variable results are found in the lower range at the value of 10200 kPa (Fig. 5h). As the flow rate of water and nitrogen increases, ENG increases as expected and this can be noticed in Fig. 5i–j respectively.

Case 4 : Maximization of Profit and Maximization of Purity

**Fig. 6:**
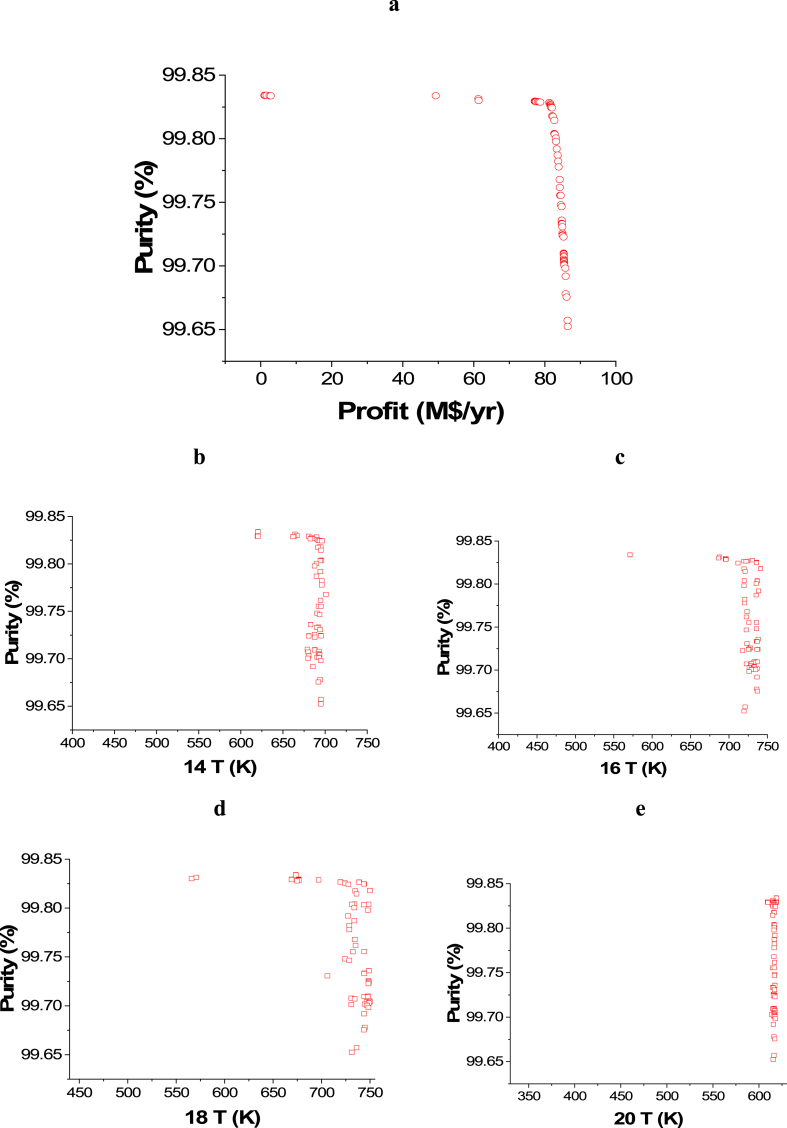

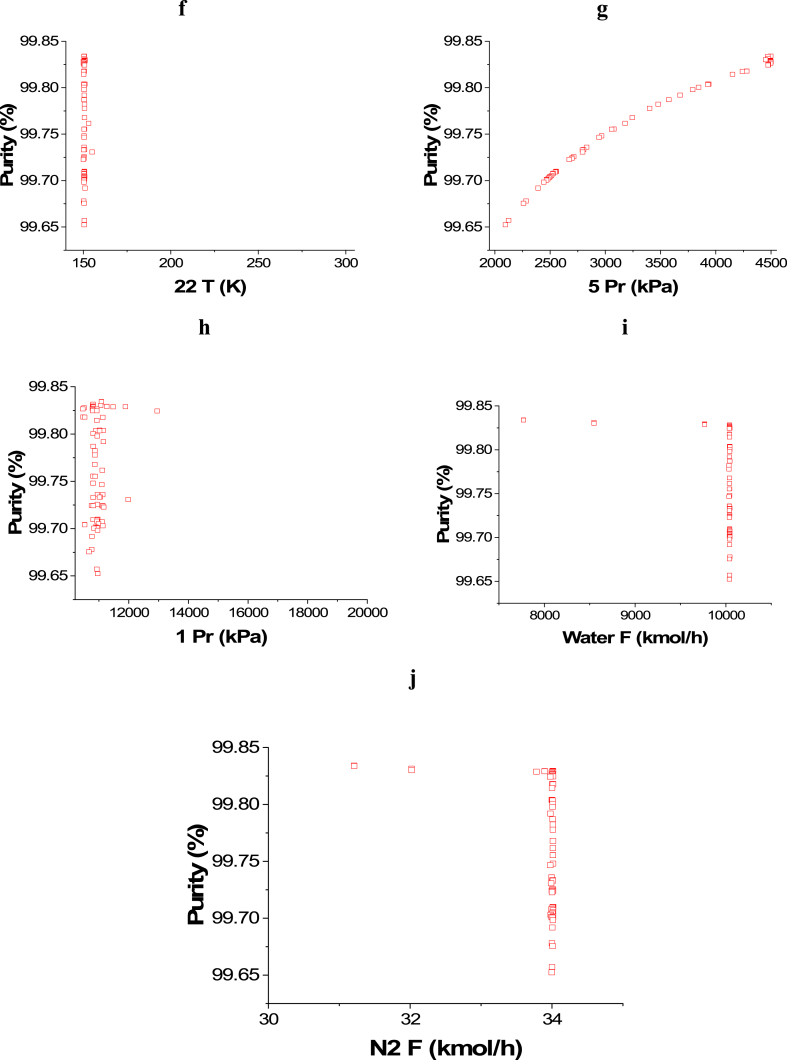
Case 4 Pareto front (a) and the corresponding decision variables (b–j) for the non-green ammonia production process (maximization of Profit and maximization of Purity).

Fig. 6a represents the Pareto front for Purity and Profit maximization and the influence of the selected decision variables is shown in Fig. 6b–j. The impact of the 14 T decision variable on the Purity objective function can be seen in Fig. 6b and it is obvious that, in general, the entire points are among the value of 700 K. This result is desirable to reach a high conversion rate and thus higher ammonia production and to minimize ENG. T 16 (Fig. 6c) and T 18 (Fig. 6d) decision variables' results are close to their highest value in the range which is 750 K. The same behavior is noticed with T 20 decision variable (Fig. 6e) as the results converged closer to the upper value in the range (620 K). To achieve the highest purity and production, T 22 decision variable results needed to converge at the lower range's value (150 K) as presented in Fig. 6f. As 5 Pr increases, the Purity objective function increases as well (Fig. 6g), however, it also raises the operating cost and therefore, lessens the Profit objective function. Decreasing the pressure of the gases is linked to a reduction in the operating cost, which increases the profit. Nevertheless, the purity is not affected in this case as the trend of the obtained results for water and nitrogen flow is different here (Fig. 6h). In this case, Water F (Fig. 6i) and N_2_ F (Fig. 6j) both converged to their higher range value, which is 10046 kmol/h for water and 34 kmol/h for N_2_. This convergence manner is required to accomplish higher production with high purity and to maximize the profit.

Case 5 : Minimization of Energy (ENG), and Maximization of Profit and Purity

**Fig. 7:**
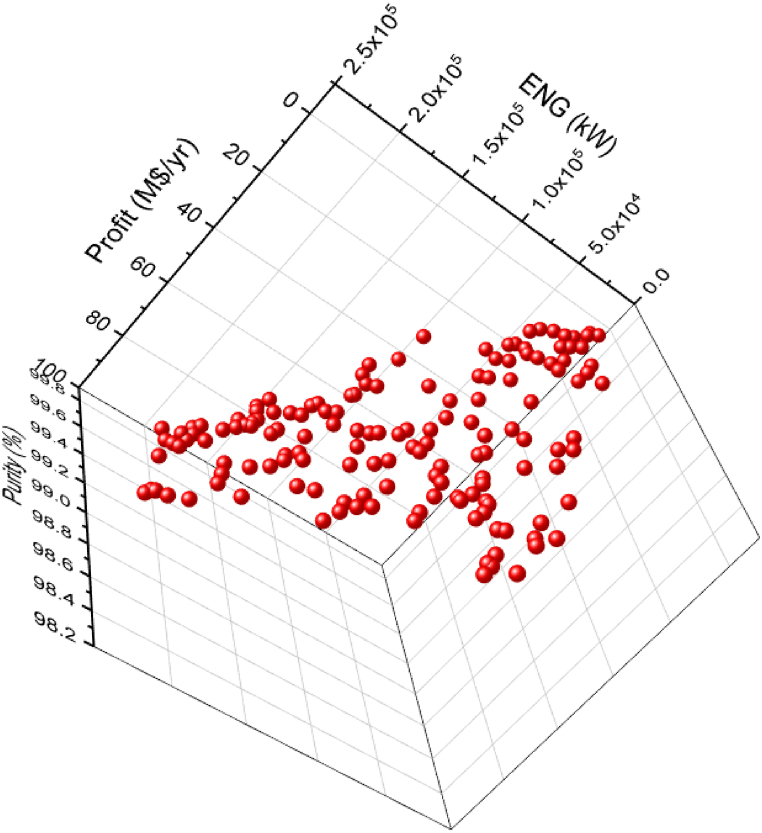
Case 5 Pareto front for the non-green ammonia production process (minimization of ENG and maximization of Profit and Purity).

**Fig. 8:**
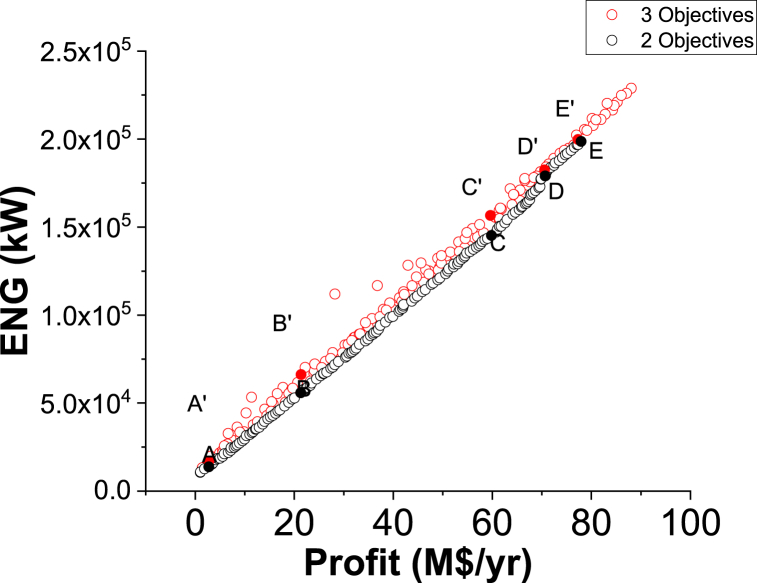
Two objectives and three objective Pareto fronts (ENG vs. Profit).

The 3D graph for the three objective case of ENG minimization and Purity and Profit maximization for the green ammonia process is shown in Fig. 7. To evaluate the three and two objective cases, Fig. 7, Fig. 8 and Table 3 and Table 4 are displayed. For a fair assessment, specific values of a chosen objective are studied.

**Table 3:** Two objective and three objective optimization results comparison for selected values of Profit from Fig. 8.

Point	Profit vs. Energy			Point	Profit vs. Energy vs. Purity		
	Profit M$/yr	Energy kW	Purity % (calculated)		Profit M$/yr	Energy kW	Purity %
A	2.75	13795.50	98.94	**A’**	2.75	16974.49	99.81
B	21.32	55901.97	98.89	**B’**	21.39	66214.81	99.67
C	59.82	145219.47	98.51	**C’**	59.62	156624.76	99.45
D	70.68	179007.97	99.11	**D’**	70.55	182497.96	99.14
E	77.92	198653.47	99.04	**E’**	77.31	199780.49	99.35

**Table 4:** Two objective and three objective optimization results comparison for selected values of Profit from Fig. 9.

Point	Profit vs. Purity			Point	Profit vs. Energy vs. Purity		
	Profit M$/yr	Purity %	Energy kW (calculated)		Profit M$/yr	Purity %	Energy kW
**A**	1.65	99.83	36007.89	**A’**	1.44	99.79	13219.65
**B**	49.28	99.83	131877.65	**B’**	49.12	98.92	125597.76
**C**	61.37	99.83	167506.27	**C’**	61.41	99.14	159529.78
**D**	77.36	99.83	209769.51	**D’**	77.31	99.35	199780.49
**E**	86.45	99.65	236085.8	**E’**	86.06	99.03	224843.62

Fig. 8 exhibits two Pareto fronts for the two and three objective cases. The three objective case provides more varied solutions compared to the two objective case, making it more practical for the decision-maker. Table 3 presents the results of the three objectives of the two and three objective cases for selected values of Profit, which are identified in Fig. 8.

As noticed in Table 3, the Purity results in the three objective case are better compared to the calculated values in the two objective case, which is reasonable. For instance, at point A, Purity is 98.94 % in the two objective case and it increased to 99.81 (A’) in the three objective case for the same Profit value of 2.75 M$/yr. Oppositely, to achieve this enhancement, the ENG objective function values are higher in the three objective case. At point B, ENG value is 55901.97 kW in the two objective case and 66214.81 kW in the three objective case (B’) for a Profit value of 21.32 M$/yr. When Profit is 77.92 M$/yr, approximately, ENG is 198653.47 kW (E) and 199780.49 kW (E’) for the two and three objective case respectively.

**Fig. 9:**
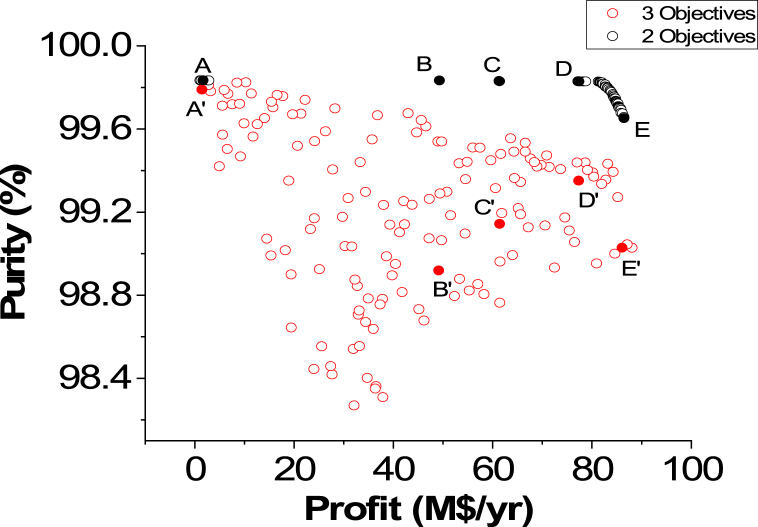
Two objectives and three objective Pareto fronts (Purity vs. Profit).

Purity vs. Profit Pareto fronts for the two and three objective cases are presented in Fig. 9. In this comparison, similar to the previous one, the three-objective Pareto front is wider in range and has more options for the decision-maker to select.

Table 4 affords the data required for investigating both cases when ENG was calculated in the two objective case and while it is one of the objectives in the three objective case. Overall, ENG values are enhanced in the three objective case in comparison to the two objective case. For instance, when Profit is approximately 49.28 M$/yr, ENG is 131877.65 kW in the two objective case. Nevertheless, it is reduced to 125597.76 kW in the three objective case. Oppositely, the Purity objective function was adversely affected in the three objective case as it was reduced from 99.83 %, in the two objective case, to 98.92 %.

### 3.2 Green ammonia process

Case 1 : Minimization of Energy (ENG) and Maximization of Profit

**Fig. 10:**
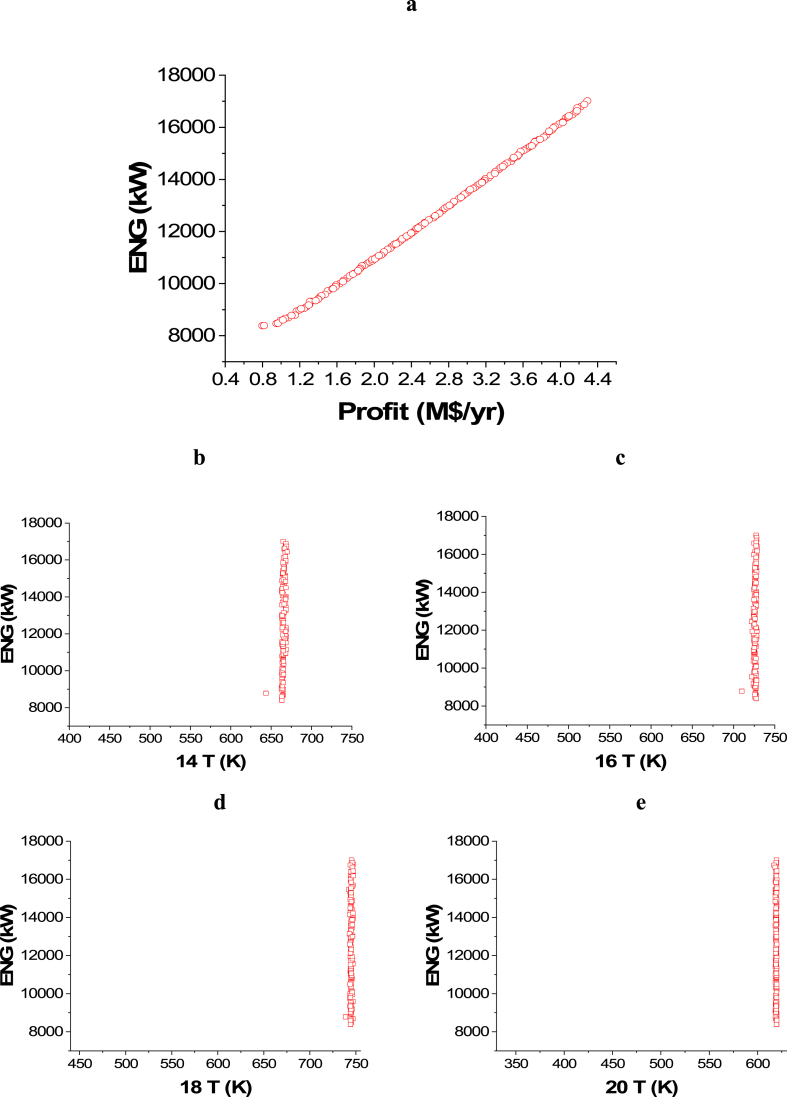

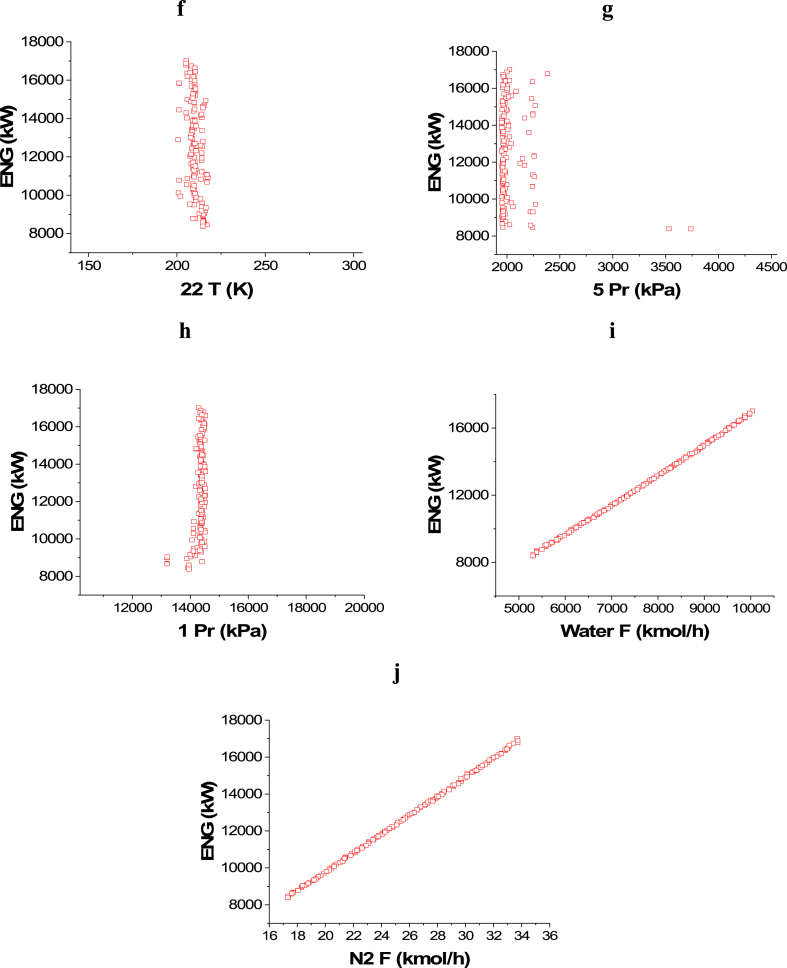
Case 1 Pareto front (a) and the corresponding decision variables (b–j) for the green ammonia production process (minimization of ENG and maximization of Profit).

The obtained Pareto front results for the case of ENG minimization and Profit maximization are presented in Fig. 10a, while the results of the corresponding decision variables are shown in Fig. 10b–j. It can be observed that the profit range in the green process is relatively less than the non-green one. This behavior is a result of being able to integrate the heat discreditsfrom the heaters to produce electricity. The effect of the 14 T decision variable on the ENG objective function can be seen in Fig. 10b and it is clear that almost all of the points converged between 644 and 670 K. Since the reaction in the ammonia reactors is an equilibrium reaction, this behavior can be attributed to the need of reaching a suitable temperature to achieve the highest conversion possible in the first reactor, which will increase the profit and minimize the energy consumption at the same time without violating any of the constraints. The T 16 (Fig. 10c) and the T 18 (Fig. 10d) decision variable results are found close to their upper range values (750 K). Minimizing ENG requires converging close to this value to reduce the cooling energy and keep the high production need to maximize the profit and the same is applied for the T 20 decision variable (Fig. 10e) since the values of the results are also close to the upper range value (620 K). For the T 22 decision variable, the results varied from 217 to 200 K as shown in Fig. 10f. To minimize energy consumption and maximize profit, less cooling is needed, but achieving the required purity and meeting the decided constraint is also essential, which explains converging around these values. Minimizing the water pumping pressure (5 Pr) is reasonable for both objectives and explains the trend seen in Fig. 10g, as the solutions converged towards the lower range value (1950 kPa). Less gas compression is also needed to serve both of the objectives, but to achieve higher production and the needed purity, it can be seen that 1 Pr decision variable solutions mostly converged between 13889 and 14525 kPa (Fig. 10h). Higher water flow (Water F, Fig. 10i) is linked to higher hydrogen production, and hence, with higher nitrogen flow (N_2_ F, Fig. 10j) more ammonia production is achievable but more ENG is required for heating, cooling, pumping, etc.

Case 2 : Maximization of Profit and Maximization of Purity

**Fig. 11:**
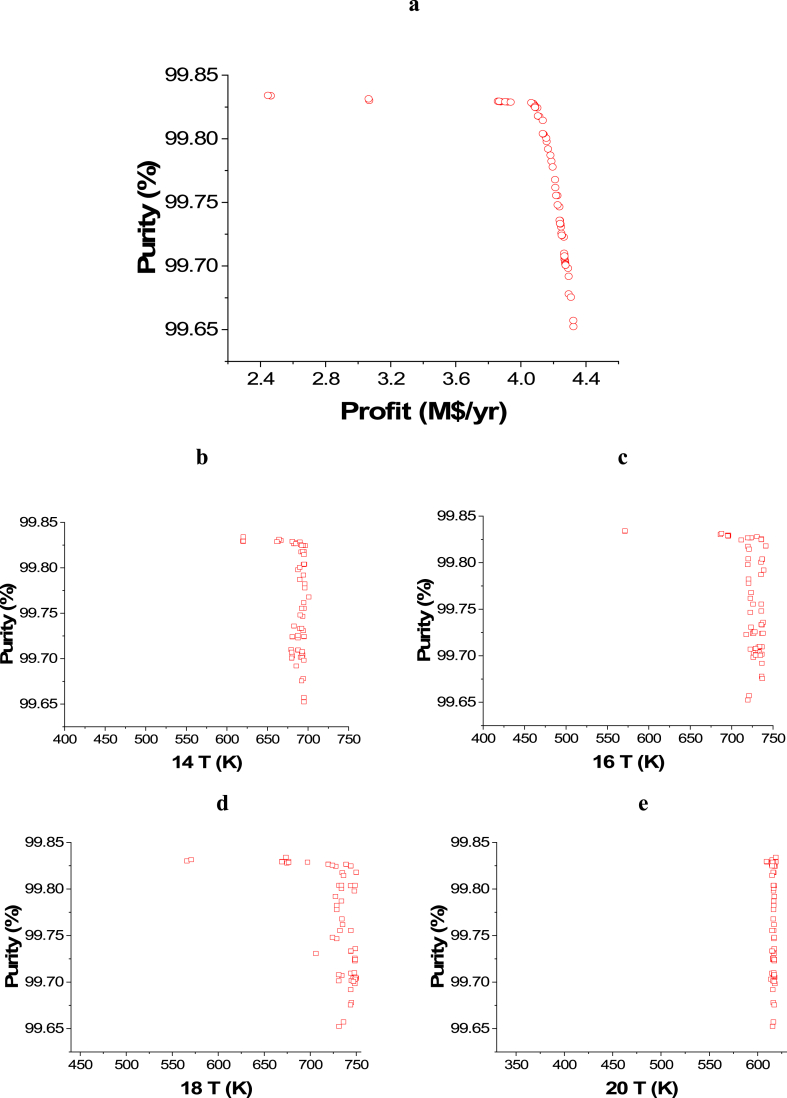

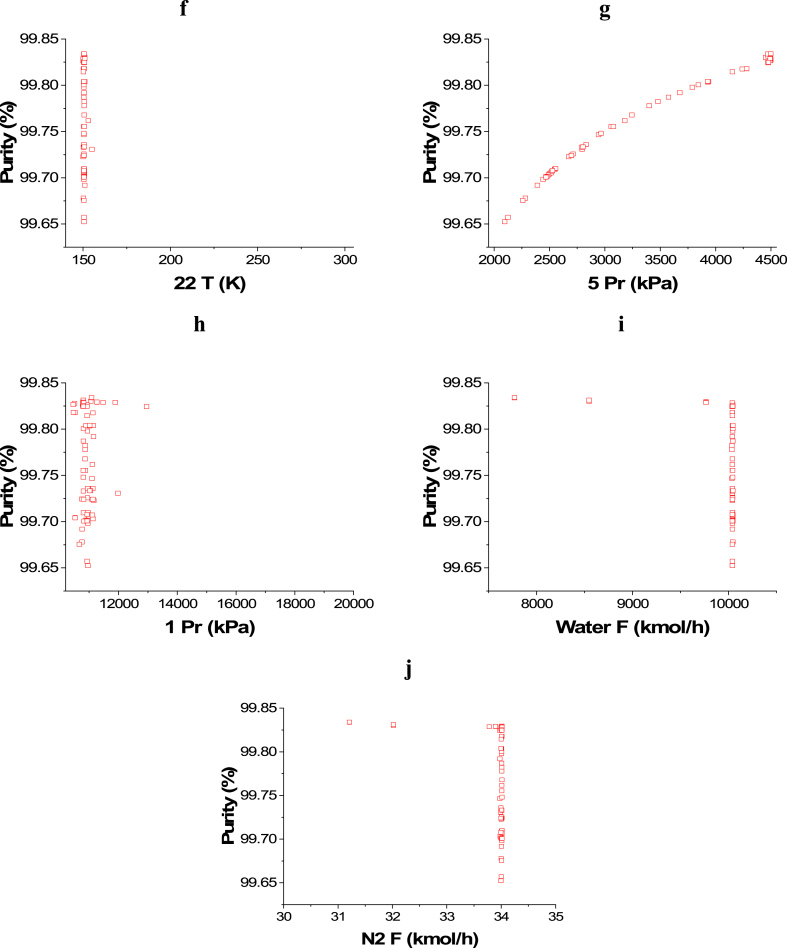
Case 2 Pareto front (a) and the corresponding decision variables (b–j) for the green ammonia production process (maximization of Profit and maximization of Purity).

The attained trade-off results for the case of Purity and Profit maximization are seen in Fig. 11a and the effects of the selected decision variables are displayed in Fig. 11b–j. The influence of the 14 T decision variable on the Purity objective function is shown in Fig. 11b and it is seen that nearly all of the points converged between around the value of 700 K. This temperature is needed to achieve a high conversion rate for higher ammonia production and to minimize ENG. T 16 (Fig. 11c) and T 18 (Fig. 11d) decision variables' solutions converged close to their upper range's value (750 K). This is also the case for T 20 decision variable (Fig. 11e) as all of the points are close to the upper range value (620 K). To achieve the highest purity and higher production, T 22 decision variable results are found at the lower range's value (150 K) as shown in Fig. 11f. Increasing 5 Pr increases the Purity objective function (Fig. 11g), but increases the operating cost and hence, minimizes the Profit objective function. Reducing the pressure of the gases is associated with reducing the operating cost and increasing the profit, but it does not affect the purity in this case since the trend of the obtained results for water and nitrogen flow is different here compared to case 1 (Fig. 11h). In this case, Water F (Fig. 11i) and N_2_ F (Fig. 11j) both converged to their higher range value, which is 10046 kmol/h for water and 34 kmol/h for N_2_. This variation of DVs is needed to achieve higher production with high purity and to maximize profit.

Case 3 : Minimization of Energy (ENG) and Maximization of Profit and Purity

**Fig. 12:**
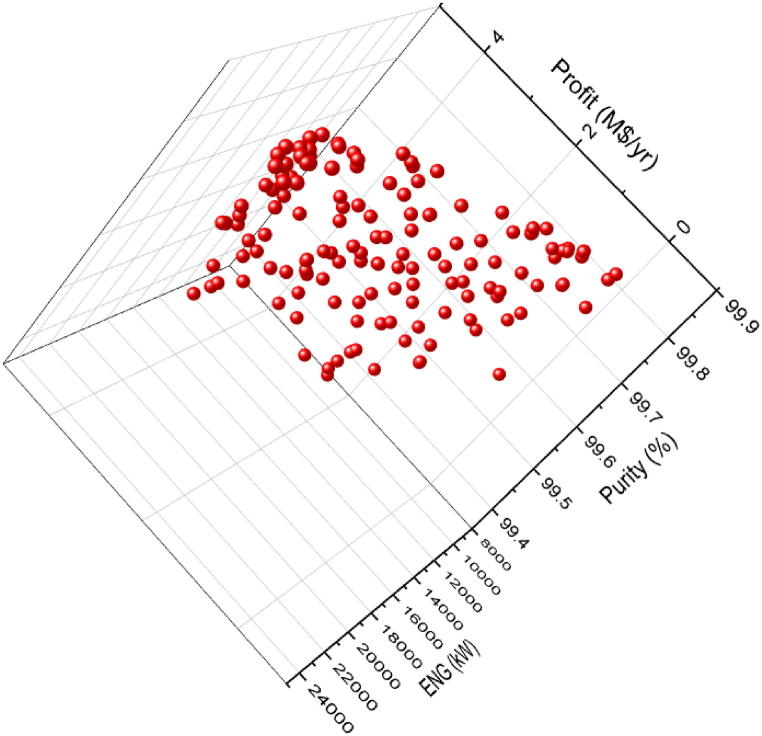
Case 3 Pareto front for the green ammonia production process (minimization of ENG and maximization of Profit and Purity).

**Fig. 13:**
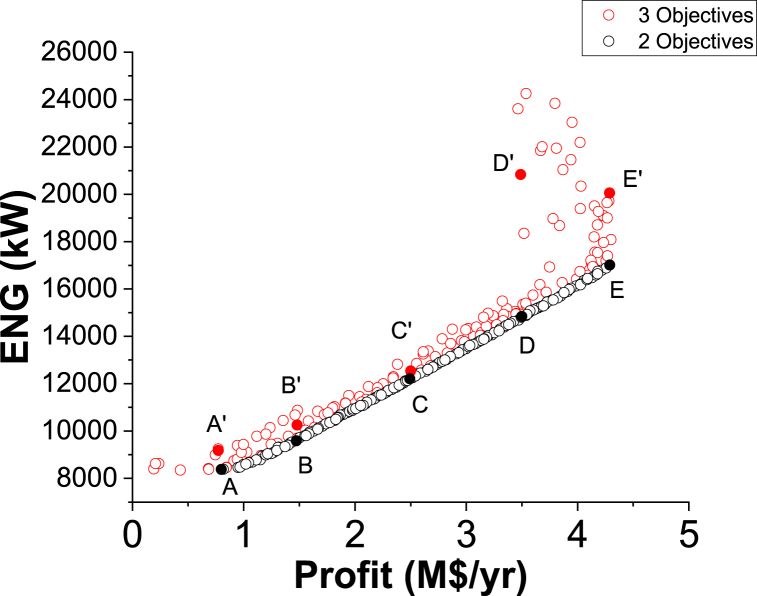
Two objectives and three objective Pareto fronts (Energy vs. Profit).

The 3D plot for the three objective case of ENG minimization and Purity and Profit maximization is seen in Fig. 12. For the comparison between the three and two objective cases, Fig. 12, Fig. 13 and Table 5 and Table 6 are presented. For a reasonable comparison, particular values of a chosen objective are selected.

**Table 5:** Two objective and three objective optimization results comparison for selected values of Profit from Fig. 11.

Point	Profit vs. Energy			Point	Profit vs. Energy vs. Purity		
	Profit M$/yr	Energy kW	Purity % (calculated)		Profit M$/yr	Energy kW	Purity %
A	0.80	8382.89	99.69	**A’**	0.77	9180.51	99.82
B	1.47	9584.44	99.52	**B’**	1.48	10253.46	99.80
C	2.49	12203.55	99.53	**C’**	2.50	12537.27	99.65
D	3.50	14840.85	99.51	**D’**	3.49	20829.72	99.83
E	4.29	17012.47	99.54	**E’**	4.29	20059.77	99.64

**Table 6:** Two objective and three objective optimization results comparison for selected values of Profit from Fig. 14.

	Profit vs. Purity				Profit vs. Energy vs. Purity		
	Profit M$/yr	Purity %	Energy kW (calculated)		Profit M$/yr	Purity %	Energy kW
A	2.38	99.83	35460.01	**A’**	2.38	99.79	12819.12
B	3.03	99.83	33229.56	**B’**	3.02	99.76	14328.04
C	3.49	99.83	20758.33	**C’**	3.51	99.70	15344.82
D	3.70	99.77	18190.25	**D’**	3.68	99.83	22005.67
E	3.81	99.64	18289.27	**E’**	3.81	99.83	21944.89

Fig. 11 demonstrates two Pareto fronts for the two and three objective cases. The three objective case gives a wider range of results and more solutions to select, which is more practical from the industrial point of view. Table 5 tabulates the values of the three objectives of the two and three objective case for selected values of Profit, which are represented in Fig. 13.

It is clear from Table 5 that Purity values in the three objective case have improved compared to the ones calculated in the two objective case, which is expected. For example, at a Profit value of 0.80 M$/yr, Purity is 99.69 % in the two objective case, which is improved to 99.82 in the three objective case for the same Profit value (A and A’). On the other hand, the cost of this improvement is an increase in the ENG objective function. For instance, at point B, ENG is 9584.44 kW in the two objective case and 10253.45 kW in the three objective case (B’) for a Profit value of 1.47 M$/yr. When Profit is 4.29 M$/yr, approximately, ENG is 17012.47 kW and 20059.77 kW for the two and three objective case respectively.

**Fig. 14:**
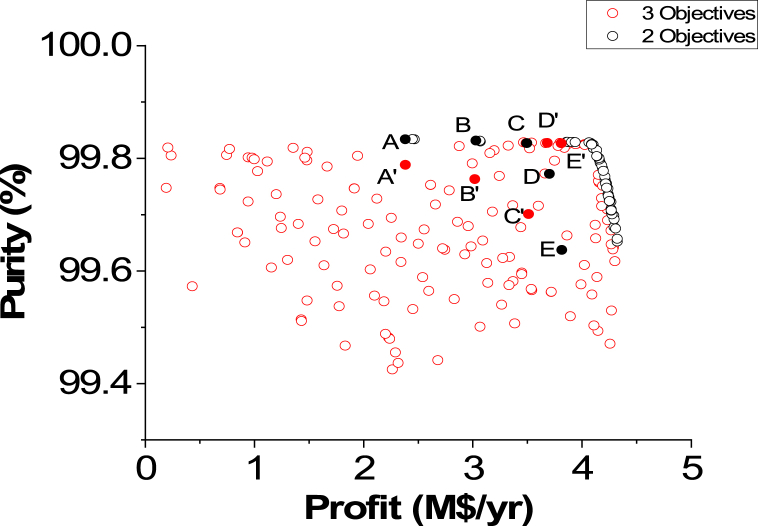
Two objectives and three objective Pareto fronts (Purity vs. Profit).

Purity vs. Profit Pareto fronts for the two and three objective cases are presented in Fig. 14. In this comparison, similar to the previous one, the three-objective Pareto front is wider in range and has more options for the decision-maker to select.

Table 6 provides the data needed for analyses for both cases when ENG was calculated in the two objective case and while it is one of the objectives in the three objective case. In general, ENG values are improved in the three objective case compared to the two objective case. For example, when Profit is approximately 2.38 M$/yr, ENG is 35460.01 kW in the two objective case. But, it is reduced to 12819.12 kW in the three objective case. On the other hand, the Purity objective function was negatively affected in the three objective case as it was reduced from 99.83 %, in the two objective case, to 99.79 %.

## 4 Conclusions

MOO study was conducted for an ammonia synthesis process using the I-MODE algorithm with Excel VBA. Two sources of energy (electricity) were considered for the same process, one of them is natural gas (non-green) and the other one is solar energy (green). The total considered objectives are minimization of CO_2_ emissions and ENG and maximization of Purity and Profit. Four two-objective cases and one-three objective case are solved for the non-green process, while two bi-objective cases and one three-objective MOO cases are solved for the green process. The non-green process emits high amount of CO_2_ gas, while the green process is considered as emission-free. The conclusions can be summarized through the following points.

• For the non-green process, it was found that CO_2_ emissions and Profit objective functions (case 1) are highly affected by nitrogen flow rate as higher electricity needs to be generated as the flow of the gas increases.
• Similarly, ENG and Profit (case 2), and ENG and Purity (case 3) cases are also affected by the flow rates of both water and nitrogen, which is also the case with the green process (case 1 and 2 respectively). On the other hand, the 5 Pr decision variable was the key factor for the conflict between Profit and ENG objectives.
• In general, the Profit in the non-green process is higher than that of the green process. For example, the Profit results in case 2 (non-green) varied from 0.7 to 80 M$/yr, while the range is 0.8–4.4 M$/yr for the same objectives studied in case 1 in the green process.
• For each process, three objective MOO cases were carried out and the results are compared with the related two objective cases. The obtained results indicated an improvement in the third solved objective compared to the two objective case calculated values. For instance, in case 3 in the green process, when the Profit value is about 0.80 M$/yr, the Purity is 99.69 % in the two objective case, but for the same Profit value it is enhanced to 99.82 % in the three objective case. This is also the case with the non-green process in case 5 as Purity is improved from 98.94 % in the two objective case and to 99.81 % in the three objective case for a Profit value of 2.75 M$/yr.
• In most of the studied cases, water and nitrogen flow rates showed a high influence on the results and caused conflict between the objectives.

## CRediT authorship contribution statement

**Ashish M. Gujarathi:** Conceptualization, Data curation, Formal analysis, Funding acquisition, Investigation, Methodology, Project administration, Resources, Software, Supervision, Validation, Visualization, Writing – original draft, Writing – review & editing. **Rashid Al-Hajri:** Formal analysis, Methodology, Software, Supervision, Writing – review & editing. **Zainab Al-Ani:** Data curation, Formal analysis, Investigation, Resources, Software, Validation, Visualization, Writing – original draft. **Mohammed Al-Abri:** Formal analysis, Supervision, Writing – review & editing. **Nabeel Al-Rawahi:** Formal analysis, Investigation, Supervision, Writing – review & editing.

## Declaration of competing interest

The authors declare that they have no known competing financial interests or personal relationships that could have appeared to influence the work reported in this paper.
